# Asymmetric transfer hydrogenation of imines and ketones using chiral Ru(II)Cl(η^6^-*p*-cymene)[(*S*,*S*)-*N*-TsDPEN] catalyst: a computational study

**DOI:** 10.1186/1758-2946-4-S1-P16

**Published:** 2012-05-01

**Authors:** Petr Kačer, Jiří Václavík, Jan Přech, Marek Kuzma

**Affiliations:** 1 ICT – Prague, Technická 5, 166 28 Prague, Czech Republic; 2 Institute of Microbiology, Academy of Sciences, Vídeňská 1083, 142 20, Prague 4, Czech Republic

## 

*Noyori et al.* in 1996 showed that the Ru(II)Cl(η^6^-*p*-cymene)[*N*-*p*-tosyl-1,2-diphenylethylenediamine] (= [RuCl(η^6^-*p*-cymene)TsDPEN]) in a HCOOH / triethylamine (TEA) mixture was able to efficiently hydrogenate substituted isoquinolines with high enantioselectivity (asymmetric transfer hydrogenation (ATH)) [1]. Almost simultaneously, the same system was reported to reduce ketones superbly by *Fujii et al.*(1996), suggesting that the mechanisms of ATH of C=N and C=O bonds should be alike [2]. The mechanism of the asymmetric reduction of ketones was extensively discussed by *Noyori* in a computational study in 2001, which proposed that the reaction proceeds *via* six-membered transition states (TSs) in the outer coordination sphere of ruthenium [3]. However, this mechanistic concept is not compatible with the ATH of imines, as pointed out by *Martins et al.* in 2009 [4]. According to the original mechanism, the (*S*,*S*)-complex would give an (*S*)-configured product, which conforms with the results for ketones but disagrees with experimental observations for the ATH of imines. The key element explaining this contrast seems to be the fact that an imine can only be reduced under acidic conditions, which supports the notion of requisite imine protonation, even though this is still not entirely confirmed. In the present work density functional theory (DFT) computational methods were used to investigate the increasingly popular ionic mechanistic concept for the ATH of imines on the Ru(II)Cl(η^6^-*p*-cymene)[(*S*,*S*)-*N*-*p*-tosyl-1,2-diphenylethylenediamine] chiral catalyst. Applying the ionic mechanism, the reaction preferentially affords the (*R*)-amine product, which is in agreement with the experimental observations. Calculated transition state structures for the hydrogenation of protonated 1-methyl-3,4-dihydroisoquinoline are discussed together with their preceding and following energy minima. Stabilization of the favorable transition state by a CH/π interaction between the η^6^-*p*-cymene ligand and the substrate molecule is explored in depth to show that both C(sp^2^)H/π is more probable than C(sp^3^)H/π in this molecular system. Finally, transition state geometries for the ATH of acetophenone are proposed, which take the “standard” six-membered cyclic form.
